# Multidrug resistance in *Pseudomonas aeruginosa*: genetic control mechanisms and therapeutic advances

**DOI:** 10.1186/s43556-024-00221-y

**Published:** 2024-11-27

**Authors:** Yuanjing Zhao, Haoran Xu, Hui Wang, Ping Wang, Simin Chen

**Affiliations:** State Key Laboratory of South Western Chinese Medicine Resources, School of Pharmacy, Chengdu University of Traditional Chinese Medicine, Chengdu, 611137 Sichuan China

**Keywords:** Drug resistance pathways, Gene regulatory network, Regulatory factors, *Pseudomonas aeruginosa*

## Abstract

*Pseudomonas aeruginosa* is a significant opportunistic pathogen, and its complex mechanisms of antibiotic resistance pose a challenge to modern medicine. This literature review explores the advancements made from 1979 to 2024 in understanding the regulatory networks of antibiotic resistance genes in *Pseudomonas aeruginosa*, with a particular focus on the molecular underpinnings of these resistance mechanisms. The review highlights four main pathways involved in drug resistance: reducing outer membrane permeability, enhancing active efflux systems, producing antibiotic-inactivating enzymes, and forming biofilms. These pathways are intricately regulated by a combination of genetic regulation, transcriptional regulators, two-component signal transduction, DNA methylation, and small RNA molecules. Through an in-depth analysis and synthesis of existing literature, we identify key regulatory elements *mexT*, *ampR*, and *argR* as potential targets for novel antimicrobial strategies. A profound understanding of the core control nodes of drug resistance offers a new perspective for therapeutic intervention, suggesting that modulating these elements could potentially reverse resistance and restore bacterial susceptibility to antibiotics. The review looks forward to future research directions, proposing the use of gene editing and systems biology to further understand resistance mechanisms and to develop effective antimicrobial strategies against *Pseudomonas aeruginosa*. This review is expected to provide innovative solutions to the problem of drug resistance in infectious diseases.

## Introduction

*Pseudomonas aeruginosa* (PA), a Gram-negative bacterium, has attracted significant attention due to its widespread presence in natural environments and its notable pathogenicity in the medical field. The bacterium's remarkable adaptability to various environments and diverse ecological niches greatly facilitates its ability to cause chronic infections in hosts. This adaptability and pathogenic versatility are primarily attributed to its large genome, which contains a complex regulatory network that allows it to respond effectively to different environmental stressors [1, 2]. In the context of this work, under the regulatory network, we will elucidate the intricate interactions between transcription factors and their directly regulated target genes, which constitute the network. These transcription factors, which are a specialized category of proteins, play a pivotal role in the regulation of gene transcription by binding to the upstream regions of genes, primarily within promoter regions, thereby facilitating or inhibiting the transcriptional process. Our investigation will concentrate on a specific subset of transcription factors that exert considerable influence under particular cellular conditions. By analyzing the activity of these transcription factors alongside the genes they regulate, we seek to enhance our understanding of the regulatory mechanisms that govern gene expression. By analyzing the activity of these transcription factors alongside the genes they regulate, we seek to enhance our understanding of the regulatory mechanisms that govern gene expression. Furthermore, it is noteworthy that resistance genes within the PA genome can acquire resistance to multiple antibiotics through mechanisms such as point mutations and genetic recombination [3]. This high capacity for mutation, coupled with the organism's extensive genome, significantly contributes to its clinical resistance, which can result in fatal outcomes in severe cases [4–6]. Documented instances of this bacterium developing resistance through various mechanisms in clinical settings include, but are not limited to, the reduction of outer membrane permeability, the upregulation of active efflux systems, the production of antibiotic-inactivating enzymes, and the formation of biofilms [7, 8]. These resistance pathways primarily encompass resistance genes and regulatory factors, including transcription factors, DNA methylation, small RNA molecules, and two-component signaling systems [9, 10]. The activation of these regulatory pathways directly contributes to the resistance of PA to a range of antibiotics and heavy metal ions [11].

The multidrug resistance exhibited by PA is supported by a sophisticated regulatory mechanism that manages genetic information. The intricate interplay among various regulatory factors and their corresponding target genes forms a regulatory network. This complexity not only illustrates the heterogeneity of drug resistance but also highlights shared characteristics and evolutionary trends in pathogenicity across different strains. Influential factors such as environmental stressors, genetic variability, and host immune responses can significantly affect the expression and regulation of resistance genes [8, 12–15]. Consequently, we will undertake a thorough review and evaluation of these regulatory networks.

In this work, we conducted a comprehensive analysis of the PA drug resistance gene information network, thereby elucidating genes and regulatory elements that are pivotal in modulating multiple drug resistance pathways. These central targets are consistent across different drug resistance profiles, providing potential avenues for the development of novel therapeutic approaches. Subsequent research will focus on validating the functions of these key genes and regulatory elements, and elucidating their mechanisms of action in bacterial adaptability and pathogenicity, thereby facilitating the development of more precise and efficient intervention strategies against drug-resistant PA.

## The impact of outer membrane permeability on PA resistance

The intracellular drug concentration of PA is closely related to its outer membrane permeability. A decrease in outer membrane permeability leads to a reduced influx of therapeutic agents into the bacterial cell, thereby diminishing the effectiveness of antibiotics. In severe cases, this reduction in permeability can facilitate the emergence of antibiotic resistance in PA. The characteristics of the outer membrane's permeability are primarily influenced by the unique architecture of its phospholipid and lipopolysaccharide bilayer, along with the presence of porin channels. Porin proteins are vital for the transport of substances into the cell and act as the main pathways for the entry of antimicrobial agents [16]. The expression of various outer membrane channel proteins in PA is intricately controlled by a complex array of regulatory factors, which establish sophisticated regulatory networks. The OprF protein in PA serves a dual function; it is essential for maintaining the integrity of the outer membrane and interacts with other outer membrane proteins, such as OprL and OprI, thereby affecting bacterial adhesion and the recognition of host signals [17]. Additionally, the fluidity of the outer membrane is a critical biophysical property that allows PA to adapt to environmental changes and maintain cellular functions [18, 19]. The regulation of porin channel expression is meticulously orchestrated by genetic regulatory networks.

### PA resistance genes associated with outer membrane permeability

We have conducted a literature review spanning nearly 38 years and identified a total of 18 genes from the opr and opd families that are closely associated with this pathway. Sorted alphabetically, these genes have been listed in Table 1.

**Table 1:** PA outer membrane pore protein genes and their corresponding PAO1 gene numbers, molecular weights, genomic location, length in the PAO1 genome, structural features, and regulatory elements that regulate the expression of these pore proteins (http://pseudomonas.com)

Gene	PAO1 Gene number	molecular weight (kDa)	location	length(bp)	manipulator substructure	Regulatory factors controlling pore protein expression	reference
oprB	PA3186	50.8	3,575,912..3577276 (-)	1365	PA*3190-3186*	Anr, GltR	[20]
oprD	PA0958	48.4	1,043,983..1045314 (-)	1332	/	ArgR, CzcR	[21]
oprE	PA0291	49.7	327,284..328666 ( +)	1383	No	IHF, OxyR	[22]
oprF	PA1777	37.6	1,921,174..1922226 ( +)	1053	/	AmpR	[23]
oprG	PA4067	25.2	4,544,607..4545305 ( +)	699	/	Anr	[24]
oprH	PA1178	21.6	1,277,006..1277608 ( +)	603	*oprH-phoP-PhoQ*	PhoP-PhoQ, BrlR, BqsR/CarR	[25]
oprM	PA0427	52.6	476,333..477790 ( +)	1458	/	MexZ	[26]
oprO	PA3280	47.8	3,673,008..3674324 (-)	1317	/	PhoB, TctD	[27]
oprP	PA3279	48.2	3,671,227..3672549 (-)	1323	/	PhoB, TctD	[28]
opdB	PA2700	47.3	3,053,844..3055151 ( +)	1308	PA*2699-2701*	/	[29]
opdC	PA0162	48.9	184,594..185928 ( +)	1335	/	/	[6]
opdF	PA0240	46.1	270,573..271838 (-)	1266	PA*0241*	/	[26]
opdH	PA0755	47	822,915..824198 (-)	1284	PA*0755-0751tctCBA*	/	[26]
opdK	PA4898	45.8	5,494,460..5495713 (-)	1254	/	/	[30]
oprL	PA4137	46.04	4,626,662..4627918 ( +)	1257	/	/	[31]
opdO	PA2113	44.3	2,323,554..2324783 (-)	1230	PA*2114-2110*	/	[32]
opdP	PA4503	53	5,038,901..5040355 ( +)	1455	PA*4501-4506*	/	[33]
opdQ	PA3038	46.7	3,400,684..3401949 ( +)	1266	/	NarXL	[33]

This information enhances our understanding of the mechanisms through which bacteria adapt to environmental changes and antibiotic pressures by regulating the expression of outer membrane proteins. Additionally, the data in the table clarifies the collaborative roles of various genes in maintaining bacterial physiological functions and highlights the importance of specific regulatory factors in the development of bacterial drug resistance. By targeting these genes to increase the permeability of PA's outer membrane and enhance intracellular drug concentrations, we can reverse PA’s drug resistance, allowing antibiotics to exert their maximum effect in treating PA infections

The full-length genome of PA, as published on https://www.pseudomonas.com/, spans 6,264,403 base pairs (bp) and encodes 5,572 proteins. A total of 1,472 proteins have been localized to the outer membrane in the PAO1 strain, with 18 genes accounting for a small proportion, particularly *oprD*, *oprB*, and *oprH*, which show promise as potential targets for drug resistance research. The Acinetobacter baumannii genome ranges from 3.6 to 4.2 Mb in size, encoding approximately 3,700 proteins, of which 2,000 are related to outer membrane proteins, with 18 genes also accounting for a small proportion. Non-pathogenic members of the Pseudomonadaceae Family possess a total number of outer membrane proteins similar to that of PAO1, with the same 18 genes accounting for a small proportion.

Upon analysis of the outer membrane proteins in PAO1, Acinetobacter baumannii, and non-pathogenic Pseudomonas, we identified significant patterns and differences that may have important implications for antibiotic resistance research. Firstly, by comparing genomic data, we found that although the number of Outer membrane proteins in these bacteria is similar, there are marked differences in sequence homology and function. For instance, the OprD, OprB, and OprH of PAO1 share over 80% homology with the corresponding outer membrane proteins of A. baumannii at the amino acid sequence level [34], indicating that these proteins are highly conserved evolutionarily. This conservation may reflect their key role in maintaining bacterial survival and adaptability. Further analysis of the function of these conserved proteins revealed that they play a pivotal role in the uptake and expulsion of antibiotics. For example, variations in the expression level of OprD in PAO1 are directly related to the bacterium's sensitivity to certain β-lactam antibiotics. This association has prompted us to further explore the structural characteristics of these proteins to determine how they affect antibiotic binding and transmembrane transport. Analysis of the expression patterns of resistance genes revealed differences in the responses of various bacteria to environmental pressures. In PA, MexZ regulates the expression of OprM by directly binding to its promoter region [35], while a similar regulatory mechanism is not yet clear in A. baumannii. This difference may explain why some bacteria exhibit varying rates of resistance development when faced with the same antibiotic pressure. Moreover, we compared the regulatory networks of resistance-related genes in different bacteria. We found that although there are some common regulatory factors, such as the AraC/XylS family of transcriptional activators, their regulatory logic and target gene sets differ among bacteria. For example, MexT in PA is a broad-spectrum activator of efflux pump systems, while its homologous factor in A. baumannii regulates a different set of genes [36]. These findings underscore the impact of the diversity and conservation of outer membrane proteins on antibiotic sensitivity in different bacteria, as well as the complexity of the regulation of resistance gene expression. Our analysis suggests that by targeting conserved outer membrane proteins and their regulatory factors, new antimicrobial strategies may be developed, potentially effective against a variety of bacteria.

### PA regulatory factors associated with outer membrane permeability

#### Transcriptional regulators

Transcriptional regulators are pivotal DNA sequences that exert influence over the expression of multiple genes in bacterial organisms. Transcriptional regulatory give rise to specific regulatory proteins, such as transcriptional activators or repressors, that control the expression of downstream genes by binding to specific sequences in the promoter region (the operon). These regulatory proteins influence various physiological functions of bacteria through direct or indirect mechanisms, including resistance to antibiotics, metabolism of nutrients, and adaptability to environmental changes [37]. The operon denotes a segment on the bacterial chromosome DNA that encompasses one or more related structural genes along with their shared regulatory regions [38]. Typically, there exists a binding site upstream of the operon where regulatory proteins produced by transcriptional regulators can attach to these specific DNA sequences, thereby positively or negatively regulating the transcriptional expression of genes situated downstream of the operon. The regulation by regulatory proteins and the operon encompasses various mechanisms: (1)Positive feedback regulation, where activator proteins bind to the promoter region of the operon, interact with RNA polymerase, and facilitate the initiation of transcription, thereby positively stimulating the expression of genes downstream of the operon [39];(2)Negative feedback regulation, where repressor proteins bind to the promoter or other regulatory regions of the operon, obstructing the binding of RNA polymerase or impeding transcription elongation, thus negatively impeding the expression of genes downstream of the operon [40];(3) Dual regulation, where the same regulatory gene can activate the expression of certain operons under different cellular conditions or signals, and can also inhibit the expression of other operons [41];(4)Cascade regulation, where one regulatory gene can govern the expression of another regulatory gene, which subsequently regulates specific operons, forming a cascade-like regulatory network [42]. In essence, regulatory genes synchronize and consolidate multiple distinct physiological processes by generating regulatory proteins that attach to specific operator DNA sequences, constituting a crucial molecular mechanism for bacteria to effectively adapt to alterations in their surroundings.

**Fig.1:**
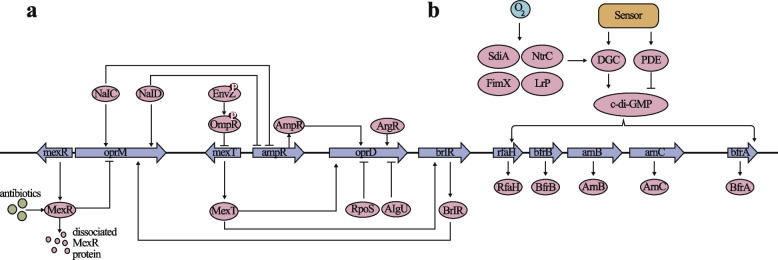
Regulatory network of outer membrane permeability in PA. DGC: diguanylate cyclases; PDE: phosphodiesterases

The OprD protein, serving as a primary antibiotic channel, is under the influence of transcriptional regulatory factors. In PA, the MexT protein activates the *oprD* gene by binding to its promoter. However, the expression of MexT itself is subject to negative feedback regulation by the EnvZ-OmpR two-component system. Upon sensing specific environmental cues (such as high osmotic pressure), the EnvZ protein undergoes autophosphorylation and subsequently phosphorylates the OmpR protein. OmpR is phosphorylated by its cognate sensor kinase, leading to a conformational change that enables it to bind DNA and act as a transcriptional regulator. Although the specific regulatory effect of phosphorylated OmpR on the *mexT* gene and the subsequent impact on *oprD* expression remain to be elucidated, it is plausible that OmpR, through its DNA binding activity, may influence the expression of genes involved in the bacterial stress response and adaptation, such as oprD. Further experimental studies are needed to delineate the precise molecular mechanisms by which OmpR modulates the expression of the mexT gene and its downstream effects on oprD and other related genes. The phosphorylated OmpR protein can attach to the promoter region of the mexT gene, inhibiting its transcription, consequently reducing the expression level of oprD indirectly [43–46] (Fig. 1a). The regulatory protein RpoS, produced by the regulatory gene rpoS, acts as a repressor that hinders the transcription of the oprD gene, resulting in enhanced resistance to carbapenems like imipenem and meropenem. AlgU, a repressor of inflammation and oxidative stress responses, also negatively regulates the oprD gene [47]. The AmpR regulatory protein is another regulatory factor capable of binding to the promoter region of the oprD gene, inducing its expression [48]. Nonetheless, AmpR itself is under negative regulation by transcription factors such as NalC and NalD, forming a complex regulatory network [49, 50]. Furthermore, the general stress response regulatory factors of bacteria are also implicated in the regulation of oprD expression. Alterations in the metabolic state can impact the expression of transcriptional regulatory factors, thereby influencing the expression of oprD [45]. In instances of aberrant bacterial metabolism, such as disorders in arginine metabolism, the ArgR transcriptional element binds to specific operator promoter regions, obstructing the binding of DNA polymerase to the promoter, safeguarding the promoter region of this operator, and ultimately boosting the transcription of genes within the operator structure. This mechanism shields the transcription of the oprD gene. The ArgR binding sequence is positioned on the antisense strand of the oprD regulatory region, centered 77 base pairs upstream of the transcription start point, arranged akin to the arc promoter, with ArgR safeguarding a 47 base pair region between the -10 and -35 regions of the oprD promoter from nuclease digestion [51]. Additionally, the cell density sensing system of PA is also involved in regulating the expression of the oprD gene. As the bacterial population density escalates, the concentration of intercellular signaling molecules rises, impeding the transcription of the oprD gene [52]. Apart from transcriptional regulation, the expression of oprD is also regulated at the post-translational level. Multidrug efflux pump systems like MexAB-OprM can influence the transcription of the oprD gene, thereby regulating the expression of the OprD protein [53].

OprM is another outer membrane channel protein in PA, mainly involved in the assembly of MexAB-OprM efflux pump system [54, 55]. The regulatory protein MexR acts as a repressor that specifically regulates the expression of the MexAB-OprM operon [56, 57]. As shown in Part a of Fig. 1, under normal circumstances, MexR binds to the promoter region of the MexAB-OprM operon, suppressing its expression. However, when the bacteria encounter antibiotics or oxidative stress, MexR dissociates, leading to the overexpression of the operon. The transcription factors NalC and NalD are two crucial regulatory factors capable of binding to the promoter region of the oprM gene, positively regulating its expression [58]. Simultaneously, NalC and NalD are also engaged in the oprD regulatory network, showcasing the interconnected regulatory relationships between these pathways. The BrlR regulatory protein is another transcription factor that attaches to the promoter region of the MexAB-OprM operon and can inhibit its expression [59]. BrlR itself is positively regulated by MexT, forming a feedback regulatory loop.

OprB, functioning as an outer membrane porin, is responsible for regulating the exchange of substances between the interior and exterior of the cell, and its function may be directly influenced by the global regulatory factors Anr and GltR [60]. Anr, acting as a transcriptional regulatory factor, can respond to environmental signals such as oxygen and nitric oxide levels, potentially directly or indirectly regulating the expression of OprB by modifying its DNA binding properties [61]. This regulation may also involve cellular respiration and energy metabolism, as the synthesis and function of OprB hinge on the cell's energy status. GltR, a transcriptional activator of the LysR family, is primarily associated with amino acid metabolism but may also indirectly impact the function of OprB through metabolic pathways [62]. OprB, Anr, and GltR constitute a complex regulatory network that impacts the adaptability, metabolism, and virulence expression of PA.

GltR may indirectly regulate the expression of OprB by modulating the availability of metabolites linked to cell membrane synthesis, thereby influencing the composition and permeability of the outer membrane. Additionally, Anr and GltR may partake in the synergistic regulation of virulence factors in PA [63]. They may influence the pathogenicity of the bacterium by regulating the expression of other virulence-related genes in conjunction with OprB [64]. Concerning environmental adaptability, Anr and GltR may aid the bacterium in adapting to the pressure of antibiotics and other harmful substances by regulating the expression of OprB and other related genes [65]. Anr and GltR may also be involved in signal transduction pathways that affect the expression of OprB [66]. Anr may respond to nitric oxide signals, while GltR may respond to metabolic signals; these signal transduction pathways are interconnected with the regulatory network of OprB, forming a finely tuned system that ensures the bacterium can effectively adapt and survive in a variable environment [67, 68].

#### c-di-GMP

Cyclic dimeric guanosine monophosphate (c-di-GMP) serves as a secondary messenger in bacterial systems, contributing to the regulation of numerous physiological processes. These processes encompass the composition and structural integrity of the outer membrane, in addition to influencing membrane fluidity [69]. c-di-GMP influences the composition and structure of the outer membrane by regulating the synthesis and assembly of lipopolysaccharides and proteins in the outer membrane [70]. It can activate the expression of genes such as *rfaH*, *arnB*, *arnC*, *pel*, *bfrA*, and *bfrB*, which are involved in the synthesis of components of the outer membrane [71, 72]. c-di-GMP can also impact the expression of outer membrane porin proteins, which are pivotal channels for regulating the transmembrane transport of substances, thereby indirectly affecting the permeability and fluidity of the outer membrane [73]. The fluidity of the cell membrane is influenced by factors such as the ratio of lipids and proteins in the lipid bilayer, the length and saturation of fatty acid chains. By regulating these factors, c-di-GMP promotes or inhibits the synthesis of specific lipids, altering the fluidity of the cell membrane. c-di-GMP can enhance the proportion of unsaturated fatty acids in the cell membrane; these fatty acid chains are more flexible, thereby increasing the fluidity of the membrane [74]. As shown in Part b of Fig. 1,the synthesis of c-di-GMP is catalyzed by diguanylate cyclases (DGCs), and its degradation is carried out by phosphodiesterases (PDEs) [75, 76]. The activity of these enzymes determines the concentration of c-di-GMP within the cell. The activity of DGCs and PDEs is regulated by various signals, including environmental factors such as oxygen levels, nutrient availability, pH, and internal signals such as quorum sensing molecules [72]. Environmental signals, such as oxygen levels, can regulate the expression of DGCs and PDEs by activating transcription factors like FimX, Lrp, NtrC, and SdiA, thereby affecting the concentration of c-di-GMP [77]. Internal signals, such as quorum sensing molecules, can directly bind to DGCs or PDEs, altering their activity, and thus promptly responding to changes in group behavior [78].

#### Two-component signal transduction systems

Two-component signal transduction systems play a critical role in the adaptive response of PA to environmental changes by modulating outer membrane permeability. These systems are prevalent signaling mechanisms in bacteria that can detect alterations in the external environment and react promptly. In PA, the PhoP-PhoQ two-component system regulatory pathway comprises the membrane-bound sensor PhoQ, the cytoplasmic response regulator PhoP, and downstream target genes like the small molecule efflux pump MexGHI-OpmD [79]. Upon sensing environmental changes such as variations in external cation concentration and pH levels, PhoQ becomes activated, leading to the phosphorylation and activation of PhoP [80]. Subsequently, PhoP governs the transcriptional expression of downstream genes [81, 82]. The PhoP-PhoQ two-component system provides resistance to diverse cationic antimicrobial peptides by regulating the lipid A modification pathway, boosts the expression of small molecule efflux pumps to enhance the extrusion capacity for small molecule drugs such as phthalic acid [83], and enhances tolerance to monovalent and divalent metal ions, thereby improving cellular activity. In specific scenarios like low magnesium ion concentration, the PhoP-PhoQ two-component system triggers the activation of the PhoQ protein and phosphorylates PhoP. The phosphorylated PhoP can bind to the promoter region of the oprD gene, prompting its expression [84].

OprH, the smallest membrane pore protein in PA with a mass of 21.6 kDa, is encoded by the oprH gene, which forms an operon with phoP-phoQ. In PA, it is established that the PhoP-PhoQ and PmrA-PmrB two-component regulatory systems are induced during Mg^2+^ starvation and directly elevate the production of OprH by PhoP-PhoQ, resulting in increased resistance to the polycationic antibiotic polymyxin B [85–87]. High concentrations of Ca^2+^ ions have a detrimental effect on PhoP-PhoQ and consequently on the expression of oprH. Additionally, Ca homeostasis is regulated by the CarSR two-component system, and the phylogenetic analysis indicates a close relationship between the CarR response regulator and PhoP, suggesting potential crosstalk between Ca^2+^ and Mg^2+^ homeostasis regulation [88, 89]. OprH plays a role in stabilizing the outer membrane by interacting with LPS, and elevated levels of Ca^2+^ or Mg^2+^ can displace OprH to reinforce the outer membrane by directly interacting with LPS, resulting in reduced OprH abundance. Building on this premise, Kreamer et al. identified a two-component system BqsSR in PA that responds to Fe^2+^ and stimulates the expression of cation tolerance genes [90, 91]. Their RNA-seq analysis demonstrated that the PhoPQ operon, encompassing oprH, is regulated by the BqsSR two-component system [91].

#### Small RNAs

Small RNA (sRNA) can also regulate PA drug resistance gene expression [92]. Trans-acting sRNAs engage with target mRNAs, thereby impacting their stability or translational efficacy. Notably, RsmA and RsmZ are two sRNAs that govern the fluidity of the outer membrane by binding to specific mRNAs, thereby regulating the expression of their target genes and subsequently influencing the composition and fluidity of the cell membrane [93]. This regulatory mechanism can alter the lipid-to-protein ratio in the cell membrane, directly impacting its fluidity. The expression of RsmA and RsmZ is subject to modulation by both environmental cues and internal signals, enabling rapid adjustments to the physical attributes of the cell membrane in response to changing environmental conditions. RsmZ plays a critical role in modulating cell membrane fluidity by interacting with mRNAs that encode cell membrane-associated proteins, thereby regulating the synthesis of these proteins through effects on mRNA stability or translational efficiency [94]. Consequently, alterations in the expression levels of specific proteins within the cell membrane can lead to changes in membrane fluidity and functionality [95]. RsmA, on the other hand, may indirectly influence cell membrane fluidity by modulating global gene expression. By regulating genes associated with the cellular stress response, such as *rpoS*, *sodA*, *sodB*, *nfsA*, and *nfsB*, RsmA may impact the maintenance of cell membrane integrity and fluidity. In response to environmental stressors like temperature fluctuations or oxidative stress, the upregulation of RsmA expression can enhance the cell's resilience to such stressors [96]. Furthermore, the regulatory functions of RsmA and RsmZ may intersect with those of other regulatory elements, potentially interacting with transcription factors or broader regulatory systems to form a complex network that collectively influences cell membrane fluidity and the overall adaptability of the cell.

## The impact of active efflux pumping mechanisms on PA resistance

The upregulation of efflux pump systems and alterations in outer membrane permeability collectively form the basis of PA's intrinsic and acquired resistance to a multitude of antibiotics. Efflux pump systems are essential to the biological processes and functions of PA, as they facilitate the removal of toxic substances, including various antibiotics, from the bacterial cell. This expulsion mechanism effectively lowers intracellular concentrations of these drugs, thereby conferring resistance to the organism. By promoting cellular homeostasis, efflux pumps also assist in the elimination of metabolic byproducts and external toxins. Furthermore, they play a significant role in the bacterium's adaptation to environmental stresses, including resistance to drugs and heavy metals, as well as evasion of host immune responses. Multidrug resistance in PA is attributed in part to overexpression of the efflux pump, which expels a variety of antibiotics. The transcriptional regulation of efflux pump activity can be influenced by environmental cues and signaling molecules, leading to the induction or inhibition of specific efflux pumps [97–99]. The gene regulatory network governing efflux systems is intricate, involving multiple regulatory factors, two-component systems, and small non-coding RNAs, with complex interactions and feedback mechanisms among different pathways.

### PA resistance genes associated with active efflux pumping systems

According to the PA genome information published on https://www.pseudomonas.com/, a total of five efflux pump systems are present. All efflux pump genes of PAO1 strains are listed in Table 2 according to alphabetical order.

**Table 2:** Name of PA efflux pumping system, gene name, PAO1 number, molecular weight, position and length in PAO1, list of regulators and functions in the efflux pumping system (http://pseudomonas.com)

Displacement pump	Gene	PAO1 Gene number	molecular weight (kDa)	location	length(bp)	regulatory factors	functionality	reference
MexAB-OprM	mexA	PA0425	41.0	472,024..473175 ( +)	1152	MexR	MexR regulates bacterial drug resistance by inhibiting the MexAB-OprM efflux pump system and responding to oxidative stress	
	mexB	PA0426	112.8	473,191..476331 ( +)	3141			[16]
MexCD-OprJ	mexC	PA4599	40.8	5,154,237..5155400 (-)	1164	MexT	MexT acts as a transcriptional activator that enhances the expression of various efflux pump systems (such as MexCD-OprJ, MexEF-OprN, etc.) by binding to their promoter regions, thereby increasing bacterial resistance to a broad spectrum of antibiotics	
	mexD	PA4598	111.6	5,151,078..5154209 (-)	3132			[100, 101]
MexEF-OprN	mexE	PA2493	45	2,808,743..2809987 ( +)	1245	MexT		
	mexF	PA2494	115.5	2,810,009..2813197 ( +)	3189			[102]
MexJK-OprM	mexJ	PA3677	40.1	4,119,270..4120373 (-)	1104	MexT		
	mexK	PA3676	113.3	4,116,188..4119265 (-)	3078			[100, 101]
MexXY-OprM	mexX	PA2019	42.1	2,211,322..2212512 (-)	1191	MexZ	MexZ is a protein that regulates efflux pump expression by inhibiting mexXY transcription, either directly or by blocking RNA polymerase access	
	mexY	PA2018	112.7	2,208,169..2211306 (-)	3138			[103] [104]

The PA genome comprises a total of 12,906 genes. The examination of the ten genes presented in the accompanying table represents only a small subset of this extensive genomic repertoire. Nevertheless, these particular genes are critical for elucidating the mechanisms underlying bacterial antibiotic resistance. Despite their limited quantity, they are essential for advancing our understanding of how bacteria develop resistance to drugs and for informing the creation of novel therapeutic approaches. The genes identified in the table are involved in the resistance mechanisms of PA, primarily by facilitating the reduction of intracellular antibiotic concentrations through efflux pump systems. Regulatory proteins such as MexR and MexT are instrumental in modulating the expression of these efflux pumps, which can significantly influence the bacterium's vulnerability to antibiotic treatment. A comprehensive understanding of the structure and function of these efflux pump systems may contribute to the development of new inhibitors that could act as adjuncts to existing antibiotics, thereby improving the effectiveness of current antimicrobial therapies. By targeting the genes that produce these regulatory factors, the function of PA's active efflux systems can be diminished, thereby enhancing the efficacy of existing antimicrobial agents.

Despite the genetic similarities among PA, *Acinetobacter baumannii*, and non-pathogenic pseudomonads, notable distinctions exist in the regulation of gene expression and the functionality of active efflux systems. These differences significantly influence their respective antibiotic resistance profiles and environmental adaptability. In PA, there is a pronounced expression of genes encoding active efflux pumps, particularly *mexA*, *mexB*, and *mexC*, in multidrug-resistant strains, which are closely associated with the emergence of carbapenem resistance. Conversely, while *A.baumannii* possesses analogous efflux pump systems, the regulatory mechanisms and gene expression patterns may diverge, resulting in differing sensitivities to efflux pump inhibitors between the two bacterial species. Both PA and *A. baumannii* are prevalent drug-resistant pathogens implicated in hospital-acquired infections; however, their resistance mechanisms and spectra differ. The resistance observed in PA is primarily attributed to the overexpression of its efflux pump systems, whereas *A. baumannii*'s resistance may involve a broader array of efflux pump systems and the production of β-lactamases. In PA, various synonymous and non-synonymous mutations may occur within the regulatory gene sequences of efflux pump operons, potentially influencing the activity of these pumps and the bacterium's resistance capabilities. For instance, mutations in the *mexR* gene can result in the overexpression of efflux pumps. Non-pathogenic pseudomonads typically do not exhibit the same level of efflux pump expression as their pathogenic counterparts, as their environmental adaptability does not necessitate the rapid expulsion of foreign substances. Given the differential expression of these genes across various bacterial species, clinical treatment strategies must be tailored accordingly. For instance, efflux pump inhibitors designed for P. aeruginosa are ineffective against *A. baumannii*, highlighting the urgent need for the development of novel antibiotics or treatment strategies to address these disparities.

### PA regulatory factors associated with active efflux pumping systems

#### Transcriptional regulators

**Fig. 2:**
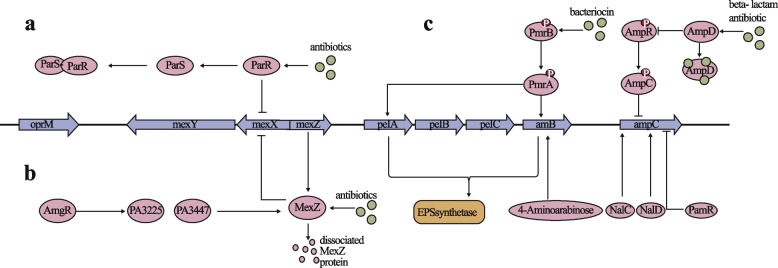
Regulatory network of active exocytosis system and antibiotic-inactivating enzyme in PA. EPS: Extracellular Polysaccharides

Efflux pump systems in PA, responsible for antibiotic resistance, are intricately regulated by transcriptional regulators. The MexAB-OprM system, a primary efflux system in PA, confers resistance to antibiotics like β-lactams and quinolones [105]. MexR, a transcriptional repressor, specifically regulates the expression of the MexAB-OprM operon. As shown in Part a of Fig. 2,MexR binds to the operon's promoter region under normal conditions, repressing its expression [106]. However, exposure to antibiotics or oxidative stress causes MexR dissociation, leading to operon overexpression. Factors like NalC and NalD stabilize MexR binding by enhancing its DNA affinity [107]. NalC and NalD, positive regulators, induce oprM gene expression and regulate MexR, forming a feedback loop. The expression of NalC and NalD is controlled by transcriptional factors like PA3225 and PA3477, highlighting the network's complexity [108]. BrlR, another regulatory protein, represses MexAB-OprM operon expression and is induced by MexT, forming a negative feedback loop [109]. AmpR, a global regulator, positively induces operon expression but is negatively regulated by NalC and NalD, showcasing cross-regulation within the network [110].

The MexXY system, another efflux pathway, is regulated by MexZ, the primary negative regulator. ParR, a transcriptional repressor, inhibits MexXY expression [111–113], but ParS binding in response to environmental signals inactivates ParR, activating MexXY expression [104, 114] As shown in Fig. 2a. MexZ binds to the MexXY operon's promoter region under normal conditions, suppressing its expression [109]. Specific environmental signals cause MexZ conformational changes (as shown in Fig. 2b), activating MexXY expression. PA5471, a newly discovered regulator, forms a heterodimer with MexZ, positively inducing MexXY expression [103, 115, 116]. PA5471 expression is negatively regulated by AmgR, forming a feedback loop.

#### Two-component signal transduction systems

The PhoP-PhoQ system positively regulates the MexEF-OprN operon, enhancing efflux system activity and bacterial resistance to certain antibiotics [117]. Under specific conditions like low magnesium ion concentration, PhoQ activates and phosphorylates PhoP, positively regulating the MexEF-OprN operon [59, 118]. MexT plays a central regulatory role, positively regulating oprD and inducing MexEF-OprN operon expression. MexT expression is negatively regulated by the EnvZ-OmpR system, indirectly reducing MexEF-OprN expression under specific environmental signals [117]. The interaction between the PhoP-PhoQ and MexT regulatory systems modulates the expression of the MexEF-OprN efflux pump, highlighting the intricate nature of antibiotic resistance regulation in PA. Understanding these regulatory mechanisms lays the groundwork for formulating strategies aimed at mitigating the activation of resistance mediated by efflux pumps.

#### DNA Methylation

DNA methylation, an epigenetic regulatory mechanism, plays a crucial role in drug resistance regulation in PA. DNA methyltransferases like Dam and CcrM methylate specific DNA sites, affecting DNA–protein binding and downstream gene expression regulation. Dam methylation impacts the MexXY efflux pump operon expression [119], while CcrM absence reduces resistance gene expression like ampC [120]. Changes in DNA methylation patterns are associated with bacterial drug resistance, with altered patterns observed in resistant strains, potentially contributing to drug-resistant phenotypes [121]. Alterations in methylation patterns are correlated with the emergence of bacterial resistance to antibiotics.

#### Small RNAs

Small RNAs like PrrF and PhrS regulate RND family efflux pumps. PrrF enhances RND pump translation [122], while PhrS inhibits RND efflux pump gene expression [123]. Cis-acting sRNAs negatively regulate downstream structural genes within resistance gene operons. For instance, armR regulates imipenemase ArmA expression [124]. The CRISPR-Cas system, initially an adaptive immune mechanism, also participates in drug resistance gene expression regulation, with Cas protein and specific crRNA inhibiting target gene expression like β-lactamase ampC [125]. These findings on the regulatory roles of small RNAs and the CRISPR-Cas system in PA suggest that targeting these regulatory mechanisms could be a promising approach to combat drug resistance.

## The impact of antibiotic-inactivating enzymes on PA resistance

PA not only diminishes the permeability of its outer membrane and enhances the expression of active efflux pump systems, but it also demonstrates intrinsic resistance to aminoglycosides and β-lactam antibiotics. This resistance is primarily due to the presence of specific modifying enzymes or hydrolases that degrade the molecular structure of these antibiotics, thereby neutralizing their efficacy and facilitating resistance [3]. The principal molecular mechanisms through which PA acquires resistance to these antibiotic classes involve aminoglycoside-modifying enzymes and β-lactamases. Furthermore, the regulatory network that oversees the antimicrobial inactivating enzymes in PA is complex, characterized by intricate interactions and cross-regulation among various enzyme classes and regulatory pathways.

### PA resistance genes associated with antibiotic inactivating enzymes

β-Lactamases represent a group of proteins with the ability to hydrolyze and render ineffective β-lactam antibiotics. PA produces various types of β-lactamases, including extended-spectrum β-lactamases, cephalosporinases, and metallo-β-lactamases. Beta-lactamases inactivate antibiotics by hydrolyzing the beta-lactam ring, a mechanism achieved through the enzymatic catalytic activity. Furthermore, These enzymes can be acquired and disseminated among bacteria through plasmid-mediated transfer or chromosomal mutations. Notable low molecular weight β-lactamase genes in this context include *ampC*, *poxB*, and *ibsA*, with *ampC* being the most prominently expressed gene in PA [126, 127]. AmpC exhibits the capability to hydrolyze a wide range of broad-spectrum cephalosporins and monobactams. Cephalosporinases, also known as AmpC enzymes, are encoded by the *ampC* gene and are highly responsive to induction. They can be categorized into inducible, constitutive, and plasmid-mediated forms. Inducible AmpC enzymes, acting as environmental sensors, are directly triggered by β-lactam antibiotics, leading to the swift emergence of drug resistance phenotypes under antibiotic pressure [128]. Conversely, constitutive AmpC enzymes offer a constant level of resistance, independent of external antibiotic concentrations, serving as an inherent defense mechanism. Plasmid-mediated AmpC β-lactamases play a pivotal role in the dissemination of drug resistance, as they are often carried by plasmids and can be horizontally transferred between different bacterial strains, rapidly escalating drug resistance levels in populations [129]. The high expression levels and limited regulatory control of plasmid-mediated AmpC enzymes empower bacteria to withstand a broad spectrum of β-lactam antibiotics, intensifying the challenge of drug resistance in clinical settings [130]. Inducible AmpC β-lactamases are typically activated in response to elevated internal concentrations of β-lactam antibiotics within bacteria. These enzymes are regulated by transcriptional factors, with AmpR acting as the repressor that governs the expression of AmpC in PA [131]. Binding of β-lactam antibiotics to AmpR alleviates its inhibitory effect, leading to the transcriptional activation of the AmpC gene.

One of the strategies employed by PA to develop resistance against antibiotics is through the utilization of antibiotic-inactivating enzymes. These hydrolytic enzymes play a crucial role in breaking down the chemical structure of antibiotics, leading to a decrease in their effectiveness and enabling the bacteria to evade the lethal effects of the drugs. The genome of PA encompasses genes responsible for the synthesis of various enzymes that inactivate antibiotics, as detailed in Table 3.

**Table 3:** List of PA antibiotic inactivating enzyme names, gene names, enzyme structures, molecular weights and functions (http://pseudomonas.com)

**Enzyme**	**Gene**	**Structures**	**molecular weight (kDa)**	**function**	**reference**
beta-lactamase	*ampC*	Contains a folded structural domain of β-lactamase	43.4	Hydrolyzes the β-lactam ring and inactivates many β-lactam antibiotics	[132]
aminoglycoside-modifying enzyme	*aacC1*	Usually contains an aminoglycoside phosphotransferase domain	30	Phosphorylation of aminoglycoside antibiotics reduces their ability to bind to bacterial ribosomes	[133]
Tetracycline exonuclease	*tetA*	Contains RND-type transmembrane transporter protein structural domains	51.2	Exclusion of tetracycline antibiotics from cells and reduction of intracellular drug concentration	[134]
macrolide phosphotransferase	*mphA*	Contains a phosphotransferase structural domain	30	Phosphorylation of macrolide antibiotics reduces their antimicrobial activity	[135]
Sulfonamide-resistant dihydropteroate synthase	*sul1/sul2*	Contains the structural domain of dihydropteroate synthase	35	Inactivation of sulfonamide drugs by competitive inhibition of dihydropteroic acid synthesis	[136]
Metallo-beta-lactamase	*blaNDM*	Contains a metallo-β-lactamase structural domain	30	Hydrolyzes the β-lactam ring and inactivates many β-lactam antibiotics including carbapenems	[137]
Beta-lactamase inhibitors	*blaZ*	Contains a β-lactamase structural domain	28	Hydrolyzed penicillin antibiotics to provide drug resistance	[138]
aminoglycoside acetyltransferase (AGAT)	*aac(6')-Ie*	Contains an aminoglycoside acetyltransferase structural domain	35	Acetylation of aminoglycoside antibiotics reduces their antibacterial activity	[139]

The table is organized according to the enzymes' contributions to antibiotic resistance and the clinical significance of the antibiotic classes they affect. Notably, beta-lactamases and metallo-beta-lactamases are of significant clinical concern due to their capacity to hydrolyze a wide range of beta-lactam antibiotics, including carbapenems. Additionally, aminoglycoside-modifying enzymes and macrolide phosphotransferases specifically target certain antibiotic classes, thereby complicating the efficacy of clinical antibiotic therapies. An examination of the molecular structures and functions of these enzymes enhances our understanding of the mechanisms by which they confer antibiotic resistance to bacterial pathogens. Beta-lactamases possess domains that facilitate the hydrolysis of the beta-lactam ring, which is integral to their resistance mechanisms. Conversely, aminoglycoside acetyltransferases modify aminoglycoside antibiotics via an acetyltransferase domain, thereby diminishing their antimicrobial efficacy. The activity and expression levels of these resistance enzymes are critical determinants of bacterial resistance profiles. The potential development of small molecule inhibitors that target these enzyme domains may restore bacterial susceptibility to currently available antibiotics. The table further includes relevant gene names, protein domains, molecular weights, and functional descriptions of the enzymes. Each resistance enzyme is encoded by one or more specific genes, highlighting the direct relationship between gene expression and protein function, which is essential for the emergence of bacterial resistance. By focusing on specific resistance genes or their regulatory sequences, it is possible to design small molecule inhibitors aimed at obstructing the expression or activity of these resistance enzymes.

In the analysis of antibiotic resistance-related genes, We employed comparative genomics to explore the diversity and functionality of antibiotic-inactivating enzyme genes in different bacteria. The comparison of the beta-lactamase gene ampC between PAO1 and Acinetobacter baumannii ATCC 17978 revealed a high degree of amino acid sequence homology, but we also discovered amino acid differences near the key active sites, leading to significant differences in their hydrolytic activity against beta-lactam antibiotics such as imipenem [140]. These minor structural differences may have a substantial impact on bacterial resistance characteristics. For the aminoglycoside-modifying enzyme aacC1, We analysis revealed a high degree of conservation of this gene across different bacteria, particularly in its phosphotransferase domain. However, variability in the C-terminal region suggests potential differences in modification efficiency [141], which may play an important role in the development of resistance to aminoglycosides. Wang W et al. demonstrated that the expression of the *tetA* gene in PA is doubled under tetracycline induction compared to Escherichia coli [142], which may underlie the enhanced resistance of PA to tetracycline. The conservation of macrolide phosphotransferase mphA in different bacteria indicates its stable performance in antibiotic inactivation, but variability in the N-terminal region may affect its recognition and modification of macrolide antibiotics [143]. Comparative analysis of the sulfonamide-resistant dihydropteroate synthase sul1/sul2 revealed variations in the Km values of these enzymes for sulfamethoxazole in different bacteria [144], indicating differences in their affinity for the substrate. Research on the metallo-beta-lactamase blaNDM indicates that this resistance gene is often associated with the Tn125 transposon, enabling effective horizontal transfer between bacteria and leading to rapid dissemination of resistance to carbapenems [145]. Analysis of the expression pattern of beta-lactamase inhibitor blaZ shows that under beta-lactam antibiotic pressure, the expression of blaZ in MRSA is significantly upregulated, whereas this upregulation is not evident in sensitive strains [146]. Distribution analysis of aminoglycoside acetyltransferase aac(6')-Ie indicates its prevalence in clinical strains resistant to aminoglycosides and its rare detection in sensitive strains [147], suggesting its significant role in aminoglycoside antibiotic resistance.

### PA regulatory factors associated with antibiotic inactivating enzymes

#### Two-component signal transduction systems

Two-component systems are prevalent signaling pathways found in bacteria, comprising a sensor protein located in the membrane and a response regulator in the cytoplasm. Within PA, specific two-component systems like AmpR-AmpC, PmrA-PmrB, and ParR-ParS are involved in regulating the expression of various genes associated with drug resistance [148].

The AmpR-AmpC two-component system is comprised of the membrane-associated sensor protein AmpR, the cytoplasmic response regulator AmpC, the target gene *ampC*, and the gene that encodes β-lactamase. β-Lactam antibiotics interact with AmpR, triggering a structural alteration and phosphorylation process. Phosphorylated AmpR subsequently activates AmpC through further phosphorylation, leading to the positive regulation of the transcriptional expression of the ampC gene [149]. The overexpression of AmpC serves as a primary molecular mechanism through which PA develops resistance to β-lactams. AmpR functions as a global regulatory protein capable of binding to the promoter region of the ampC gene, thereby stimulating its expression. However, in the absence of β-lactam antibiotic induction, AmpR exhibits limited DNA binding capacity, resulting in the maintenance of ampC gene expression at baseline levels [150]. Upon exposure to β-lactam antibiotics, certain drug molecules bind to AmpD, causing its inactivation. Inactivated AmpD loses its ability to negatively regulate AmpR, leading to the activation of AmpR, which extensively binds to the *ampC* promoter region, thereby rapidly inducing high levels of *ampC* expression. NalC/D and PamR are regulatory factors that positively and negatively regulate the expression of the *ampC* gene, respectively. NalC/D can bind to the *ampC* promoter region to induce its expression, while PamR acts as a repressor inhibiting *ampC* expression (Fig. 4). The expression of NalC/D and PamR is under the control of other regulatory factors such as PA3225 and PA3477, forming a complex regulatory network [151–155]. The CreB/C/CreBC-Lon protease system involves regulatory proteins CreB/C that interact with AmpR. The CreBC complex enhances the DNA binding ability of AmpR, thereby increasing *ampC* expression levels. The Lon protease specifically degrades CreB/C, leading to a reduction in *ampC* expression.

The PmrA/B two-component system regulates PER-1 β-lactamase expression in bacteria in response to specific environmental cues such as low Mg^2+^ or high Fe^2+^.Activation of PmrB leads to the phosphorylation of PmrA, which can then bind to the promoter region of the per-1 gene, stimulating its expression. The presence of the PmrD protein, structurally similar to PmrA, can form a heterodimer with phosphorylated PmrA, inhibiting its DNA binding ability and consequently repressing per-1 expression, thus establishing a negative feedback loop. Additionally, the PmrA/B system can positively influence biofilm formation by upregulating genes encoding enzymes involved in EPS synthesis, such as *pel/psl*. Furthermore, this system can enhance the cationicity of lipopolysaccharides by activating the arnB-pmrE operon [156], leading to the incorporation of cationic groups like 4-aminoarabinose [157], thereby increasing resistance to polymyxins (as shown in Fig. 2c),. The PhoP/Q two-component system is another regulatory pathway that interacts with the PmrA/B system. Activation of the PhoP/Q system under low magnesium ion concentrations can indirectly induce the expression of *per-1* by positively regulating the PmrA/B system.

The ParR-ParS two-component system functions by detecting specific environmental cues within bacteria through the ParS sensor, which then transmits these signals to ParR via phosphorylation. Activated ParR regulates the expression of various downstream operons.This regulatory activity influences physiological processes in bacteria, including motility, resistance to drugs, virulence expression, metabolic adaptation, and the coordination of group behaviors. Notably, ParR can positively impact bacterial movement and chemotaxis, activate resistance genes like β-lactamase and oxidoreductase, modulate the expression of virulence-associated secretion systems and toxins such as hemolysin and pyocyanin, and potentially participate in the detection of group signals to regulate biofilm formation [158].

#### Transcriptional regulators

Transcriptional activators are a group of proteins that boost the transcription process of specific genes or gene clusters. They are essential for regulating gene expression within cells. AmpR is a player in conferring resistance to β-lactam antibiotics. In response to an attack by these antibiotics, AmpR can trigger the activation of the *ampC* gene [159], which codes for β-lactamase enzyme capable of breaking down β-lactam antibiotics, thus providing resistance [160]. The function of AmpR is modulated by other proteins like AmpD, enabling bacteria to finely adjust the expression of resistance genes in response to changes in their external surroundings [161].

In PA, there exist local regulatory components such as promoters, terminators, and operators that finely control the transcription of specific genes. Various cis-acting elements located upstream of the promoter, including the -35/-10 box and UP elements [162], influence the binding affinity of RNA polymerase and the efficiency of transcription initiation, thereby regulating the expression levels of downstream genes. The promoter region of the *ampC* gene, which encodes the AmpC β-lactamase enzyme, contains multiple regulatory elements: ampC-p2 serves as the primary promoter, while *ampC*-p1, p3, and p4 act as secondary promoters, collectively governing the expression of the ampC gene [131]. Mutations at specific points or induction by antibiotics can alter the functionality of these promoters. In certain operons containing drug resistance genes, terminators prematurely halt transcription to produce short RNAs upstream, and operators bind to specific proteins to inhibit the expression of downstream structural genes, contributing to the precise regulation of drug resistance gene expression. Research has demonstrated that PrrH enhances the virulence of PA by interacting with the coding sequence region of ExsA, the primary regulatory protein of the type III secretion system [163].

PA possesses a diverse array of transcriptional activators, including RamA, RamB, and PhoP, among others, which play a role in modulating the transcription of downstream genes through interactions with the promoter regions of target genes. RamA, a well-characterized transcription factor classified within the AraC family, is known to bind to the promoter regions of approximately 20 operons, thereby positively influencing the expression of multiple drug resistance genes, including various efflux pump genes, membrane permeability genes, and redox-related genes [164]. PhoP serves as a pivotal transcriptional factor involved in regulating tolerance to small molecule quinolone antimicrobials and heavy metal ions, functioning by positively modulating processes such as small molecule efflux systems and lipopeptide biosynthesis [165]. RamB, another regulatory protein, exhibits the ability to bind to specific DNA sequences and can either enhance or suppress the transcriptional expression of numerous genes. Notably, RamB is capable of upregulating the expression of genes associated with resistance mechanisms, such as the RND family gene of the antibiotic efflux pump system gene mexAB-oprM, the β-lactamase *ampC* gene, and the aminoglycoside-modifying enzyme gene, thereby contributing to the regulation of antibiotic resistance in PA. Furthermore, RamB can also promote the expression of virulence factor genes linked to cell invasion and pathogenicity, including the type III secretion system, lysozyme, pyocyanin, and the virulence protein PrfX. Additionally, RamB plays a role in positively regulating the expression of biofilm genes psl and pel [166], thereby impacting the synthesis and maturation of the biofilm structure [167].

Apart from the regulatory factors previously discussed, PA exhibits metabolic global regulatory mechanisms that influence the expression of drug resistance genes indirectly. One such mechanism involves the CbrA/CbrB system, which governs the metabolic process of carbon source utilization, impacting redox balance and consequently modulating the expression of drug resistance genes controlled by SoxR [168].

## The impact of biofilms on PA resistance

The development of biofilms by PA is a sophisticated process that involves the precise control of numerous genes. Biofilms are bacterial structures that form on solid surfaces and are surrounded by a matrix of extracellular polysaccharides [169]. This compact arrangement can impede the effective diffusion of antibiotics into the biofilm, leading to a decrease in the bactericidal efficacy of antibiotics against the bacteria residing within the biofilm [76]. As a result, the bacteria demonstrate an increased resistance to pharmacological treatments and an enhanced capacity for environmental adaptation.

### PA resistance genes associated with formation of biofilm

Gene regulation is fundamental to the development of the biological periplasm and encompasses multiple levels of regulatory mechanisms, including the quorum sensing (QS) system and transcriptional regulation. QS sensing, a critical regulatory mechanism in PA, relies on acyl-homoserine lactone signaling molecules like 3-oxo-C12-HSL and C4-HSL [170]. Regulatory factors suc [171]has LasR and QscR respond to these signaling molecules, thereby activating or inhibiting the expression of a range of virulence-related genes [172]. For instance, LasR can trigger the activation of virulence factors such as AprA [173], alkaline protease, and elastase LasB, which aid bacteria in invading host tissues, evading immune responses, and acquiring essential iron ions [174]. Apart from quorum sensing, the regulatory network of PA also encompasses responses to environmental stresses, including oxidative stress responses. This involves the upregulation of antioxidant enzymes, which are under the control of regulatory factors like RhIR and Rhll [175]. Additionally, the GacA/PprB two-component system plays a crucial role in regulating quorum sensing and the expression of virulence factors [176].

We have summarized the literature from nearly 30 years and identified 13 genes related to the biofilm of PA. These genes have been sorted based on the significance of their functions and are depicted in Table 4. The full-length genome of PA, as published on https://www.pseudomonas.com/, spans 6,264,403 bp and encodes 5,572 proteins. A total of 1,278 proteins have been localized to the biofilm-associated proteins in the PAO1 strain, with 14 genes accounting for a small proportion, which may hold promise as potential targets for drug resistance research.

**Table 4:** List of PA bioepitope-related gene names, PAO1 numbers, molecular weights, positions in PAO1, lengths and functions (http://pseudomonas.com)

**Gene**	**PAO1 Gene number**	**molecular weight (kDa)**	**location**	**length (bp)**	**function**	**reference**
*algU*	PA0762	22.2	831,301..831882 ( +)	582	Regulates genes and affects a variety of physiological processes such as polysaccharide synthesis	[177]
*lasR*	PA1430	26.6	1,558,171..1558890 ( +)	720	quorum sensing induction regulator, regulates genes related to bioepithelial membrane formation	[178]
*rhlR*	PA3477	27.6	3,889,925..3890650 (-)	726	quorum sensing induction regulator, synergistic with lasR	[178]
*bfmR*	PA4101	28	4,585,149..4585889 ( +)	741	Regulation of extracellular polysaccharide synthesis and biofilm formation	
*pelA*	PA3064	104.9	3,431,045..3433891 (-)	2847	Involved in the synthesis of the extracellular polysaccharide Pel	[179]
*pelB*	PA3063	135.1	3,427,486..3431067 (-)	3582	/	
*pelC*	PA3062	18.7	3,426,928..3427446 (-)	519	/	
*pslA*	PA2231	54.8	2,453,667..2455103 (+)	1437	Involved in the synthesis of the extracellular polysaccharide Psl, which affects the stability of biological periplasmic membranes	[180]
*pslB*	PA2232	53.5	2,455,103..2456569 (+)	1467	/	
*pslC*	PA2233	33.6	2,456,569..2457480 (+)	912	/	
*algD*	PA3540	47.6	3,962,825..3964135 (+)	1311	Enzymes in the extracellular polysaccharide alginate synthesis pathway	[181]
*algT*	PA0762	22.2	831,301..831882 (+)	582	Transcription factor that regulates alginate synthesis	[182]
*mexS*	PA2491	36.8	2,806,350..2807369 (-)	1020	Involved in regulating the expression of multidrug efflux pumps	[136]

Table 4 is arranged based on the genes' functions and their physiological roles inP. aeruginosa PAO1. The table initially lists regulatory genes and transcription factors that affect a variety of physiological processes, such as algU, followed by regulators involved in quorum sensing, lasR, and rhlR. Subsequently, the table includes genes involved in the synthesis of extracellular polysaccharides, *bfmR, pelA, pelB, pelC, pslA, pslB, pslC, algD,* and *algT*, which are mainly involved in biofilm formation and bacterial adhesion. Lastly, *mexS* is included, which is involved in the regulation of multidrug efflux pump expression. The functional analysis of these genes indicates that they play a central role in the physiological adaptation, biofilm formation, quorum sensing, and development of drug resistance in bacteria. By designing inhibitors targeting the quorum sensing system, we can inhibit bacterial quorum behavior, thereby reducing their virulence and ability to form biofilms.

An examination of the quorum sensing system in PAO1 has prompted a particular focus on the regulatory factors *lasR* and *rhlR*. By comparing their homologous genes in other bacteria, such as Acinetobacter baumannii, We found that although *lasR* and *rhlR* are highly conserved across different bacteria, they exhibit significant differences in the selectivity of target gene regulation and the intensity of signal response. For instance, *lasR* in PA demonstrates a more complex regulatory pattern in controlling genes related to biofilm formation, while *rhlR* is more active in the expression of virulence factors [183]. Such differences may be related to the environmental adaptability and pathogenic mechanisms of different bacteria.

In relation to extracellular polysaccharide synthesis, an analysis of the *pel*, *psl*, and *alg* gene clusters in PA was conducted, with comparisons made to their homologous genes in other bacteria. The findings indicate that these gene clusters are pivotal in biofilm formation, and the polysaccharide synthases and transport proteins they encode are highly conserved among various bacterial species. However, variations in the promoter regions and regulatory elements of these genes across different bacteria result in diversity in biofilm formation efficiency and polysaccharide composition. Concerning multidrug resistance, a specific investigation into the expression regulation mechanism of the *mexS* gene in PA revealed that, in comparison to its homologous genes in other bacteria, such as Escherichia coli, the regulatory network governing *mexS* in PA is more complex, involving a greater number of transcription factors and signaling molecules. [184]. This complexity may significantly contribute to the elevated levels of drug resistance observed in clinical settings.

Through these comparisons and analyses, we concluded that although different bacteria have conserved genes and regulatory mechanisms in quorum sensing systems, extracellular polysaccharide synthesis, and multidrug resistance, the differences in regulatory details and efficiency have a significant impact on bacterial adaptability and pathogenicity. These findings provide important molecular targets for the development of new antimicrobial strategies.

### PA regulatory factors associated with formation of biofilm

#### Quorum sensing system

Quorum sensing (QS) system is a bacterial resistance mechanism that relies on cell density to transmit signals and coordinate the behavior of bacterial populations by producing and responding to self-inducing molecules [185]. In PA, the primary QS system is the LasI-LasR system, which governs biofilm formation by controlling the expression of downstream genes. This system involves two genes, *lasI* and *lasR*. The *lasI* gene is responsible for synthesizing the signal molecule N-acylhomoserine lactone (AHL), the autoinducer in the QS system [186]. The lasR gene encodes the LasR protein, a transcriptional activator that binds specifically to the AHL signal molecule [187]. Upon reaching a certain concentration in the bacterial population, AHLs bind to the LasR protein, activating its transcriptional function. The resulting LasR-AHL complex then binds to the promoter region of target genes, regulating the expression of downstream virulence genes such as *lasA*, *lasB*, *aprA*, and *rpoS*, and influencing the production of virulence factors [188]. These target genes typically encode proteins associated with bacterial virulence, biofilm formation, drug resistance, and cellular behavior.

**Fig.3:**
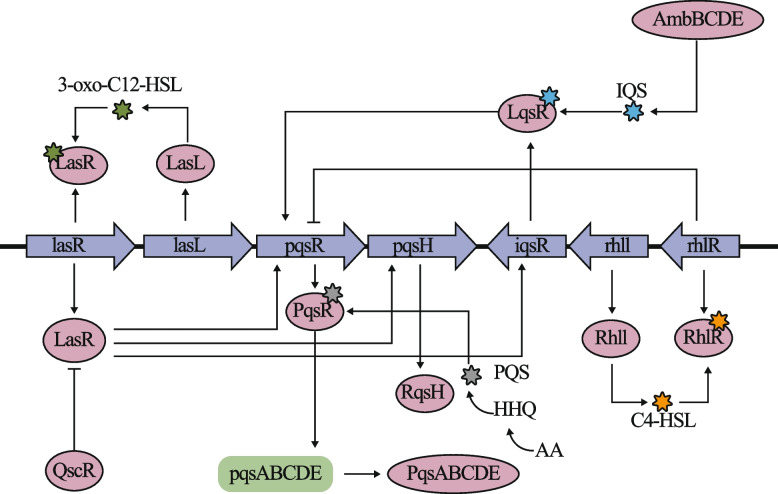
Regulatory network of QS systems in PA. IQS:2-(2-hydroxyphenyl)-thiazole-4-carbaldehyde; PQS:2-heptyl-3-hydroxy-4(1H)-quinolone; HHQ:2-heptyl-4-hydroxyquinoline; AA: anthranilic acid; C4-HSL:N-butyrylhomoserine lactone

PA possesses three additional quorum sensing (QS) systems in addition to the Las system, specifically the RhlI-RhlR, PqsABCDE-PqsR, and AmbCDE-IqsR systems. [188]. Each of these systems comprises a synthase responsible for generating a small signal molecule (autoinducer) and a transcriptional receptor protein that becomes activated upon reaching a specific concentration of the autoinducer. These systems collaborate with the Las system to regulate the production of numerous factors crucial for the growth and virulence of PA. LasR, a pivotal component in the regulatory hierarchy, can trigger downstream QS systems, which in turn can mutually enhance each other through a cascade effect. Even in scenarios where LasR function is hindered, the activation of these alternative QS systems can induce the expression of the lasB gene. Mutations in the *lasR* gene may result in diminished expression of LasB and other virulence factors under LasR regulation. Nevertheless, certain strains with *lasR* gene mutations have demonstrated LasR's ability to stimulate LasB expression via the RhlR system. RhlR, which responds to its corresponding QS autoinducer N-butyrylhomoserine lactone (C4-HSL) [189], governs the synthesis of rhamnolipids(as shown in Fig. 3).

The activated complexes of LasR and RhlR can both bind to the las-rhl box within the promoter region of the *lasB* gene, thereby directly amplifying the transcriptional activity of *lasB*. Furthermore, additional QS systems like the PqsR system can positively influence the functions of the RhlR and IqsR systems by responding to their specific QS autoinducers, such as 2-heptyl-3-hydroxy-4(1H)-quinolone (PQS) [190, 191]. The PqsR system initiates from tryptophan (Trp) and enzymatically converts it to anthranilic acid (AA), which is further transformed into 2-amino-3-ketoadipate (AK) and then synthesized into 2-amino-3-hydroxyquinoline (AHQ). The PqsABCDE complex converts AHQ to HHQ, which is subsequently oxidized to PQS by PqsH. On the other hand, the IqsR system responds to a different QS autoinducer, 2-(2-hydroxyphenyl)-thiazole-4-carbaldehyde (IQS), and positively regulates the expression of PqsR [192].

#### Transcriptional regulators

The expression of biofilm-associated genes in PA is controlled by a network of transcription factors, which act as either activators or repressors by binding to specific promoter regions of target genes. Alginate, a major component of bacterial biofilms, is synthesized by a group of genes within an operon. Among these genes, *algD* encodes the essential enzyme GDP-mannose dehydrogenase for alginate synthesis and exhibits robust transcriptional activity as the first gene in the operon [177]. In PA, the alg genes are categorized into synthesis, regulatory, and conversion genes [58]. While *algD* and *algG* directly participate in alginate synthesis, *algZ*, *algR*, and *algB* are involved in its regulation, and conversion genes such as *algT*, *mucA*, *mucB*, *mucC*, and *mucD* facilitate the transition to a mucoid phenotype.

The activation of GDP-mannose dehydrogenase encoded by the *algD* gene is a critical step in alginate synthesis regulation, controlled by both alginate conversion and regulatory genes [187]. The sigma factor encoded by *algT* positively influences its own promoter and the expression of regulatory genes like *algR*, whereas muc genes encode factors that inhibit *algT* expression [193]. The cooperative action of *algR* and *algT* leads to the upregulation of *algD* and downstream synthesis genes [194]. Mutations in muc negative regulatory genes are responsible for the increased production of alginate in mucoid strains, resulting in heightened expression of *algT* and subsequent transcription of *algR* and *algD*, leading to enhanced bacterial resistance to drugs [195].

## Drug resistance association network construction and potential targets analysis

**Fig. 4:**
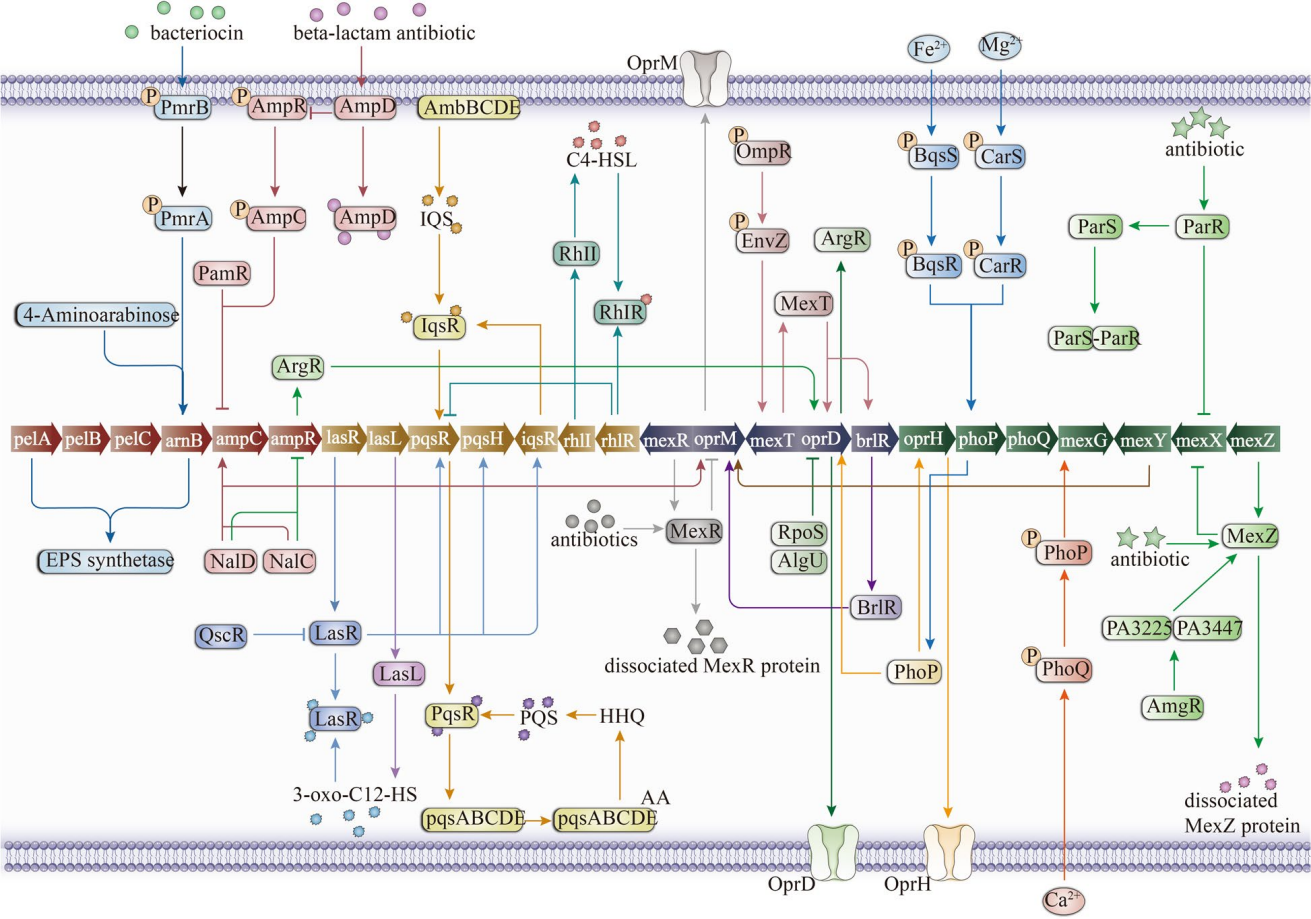
Regulatory network of drug resistance in PA

The phenomenon of drug resistance in PA is a multifaceted characteristic influenced by various factors and mechanisms, notably the outer membrane's barrier function, the intracellular efflux pump system, generation of antibiotic-inactivating enzymes and biofilm formation. The outer membrane's low permeability restricts antibiotic entry, while the upregulation of efflux pump systems like MexAB-OprM expedites antibiotic expulsion, diminishing drug accumulation within the cell [106]. The bacterium can produce hydrolytic enzymes, deactivating antibiotics and reinforcing resistance. Moreover, PA can create biofilms, which offer added protection, impede antibiotic penetration, and bolster resistance to environmental pressures. These resistance mechanisms involve a network of resistance genes and intricate regulatory processes(Fig. 4).

The identification of critical regulatory targets within this network may pave the way for the development of strategies aimed at addressing multidrug resistance in PA. These targets may encompass crucial components of efflux pumps, enzymes crucial for biofilm synthesis, and proteins that govern resistance gene expression. Disrupting these targets could potentially impede resistance development, enhance antibiotic permeability, and amplify the efficacy of current antimicrobial therapies.

MexT functions as a positive regulatory factor in reducing drug resistance by promoting the transcription of *oprD*, the outer membrane channel protein.When the concentration of carbapenem antibiotics increases in PA, the MexT protein dissociates, leading to reduced expression of the OprD outer membrane channel protein. This decrease in expression results in decreased permeability of the PA cell outer membrane, contributing to drug resistance. The ArgR regulatory factor binds specifically to the regulatory region upstream of the *oprD* gene, protecting it from nucleases and regulating its transcriptional expression [51]. Conversely, RpoS and AlgU act as negative regulatory factors suppressing *oprD* expression [196]. The two-component systems BqsSR and CarSR are phosphorylated in the presence of Fe^2+^ and Mg^2+^, influencing the expression of OprH.The ampC operon, comprising the *ampC* structural gene and its upstream regulatory DNA segment, plays a crucial role among antibiotic inactivation enzymes. The *ampC* structural gene encodes the AmpC enzyme protein, which can hydrolyze and degrade various β-lactam and some cephalosporin antibiotics [197]. Exposure to β-lactam antibiotics triggers signal cascade reactions, activating regulatory factors like AmpR [162], which upregulate the transcriptional expression of the entire *ampC* operon. This leads to increased AmpC enzymes [198], enabling bacteria to hydrolyze antibiotic molecules and develop drug resistance.The expression of AmpC is finely regulated by multiple factors, including AmpR/AmpD and NalC/NalD. AmpR, a transcriptional activator, releases signaling molecules when bacterial cell wall synthesis is hindered, activating and inducing *ampC* expression. Conversely, AmpD can inhibit AmpR activity by binding to it. Deletion mutations in *ampD* induced by β-lactam drugs promote AmpR activation and *ampC* expression. NalC/NalD, acting as transcriptional repressors, inhibit *ampC* transcription by binding to its upstream operator region [110].

The PmrA/PmrB two-component system is activated under signals like low magnesium ions, with PmrA suppressing the *arnB* gene and genes related to EPS modification enzymes such as *pelABC* upstream [199]. The multidrug resistance of PA involves various drug pump systems regulated by specific factors like MexR, NalC/D, BrlR, MexT, and MexZ [200]. These factors recognize and transmit environmental stimuli and metabolic signals, finely regulating the expression levels of target genes, forming a complex regulatory network to dynamically regulate drug resistance in PA.

The formation of biofilms by PA is associated with four QS systems, including the LasR system, RhlI-RhlR system, PqsABCDE-PqsR system, and AmbCDE-IqsR system. However, it is primarily linked to the Las quorum sensing system, comprising the LasR and LasI genes, which regulate the expression of pathogenic genes and virulence factor production through 3-oxo-C12-HSL [187].

**Fig. 5:**
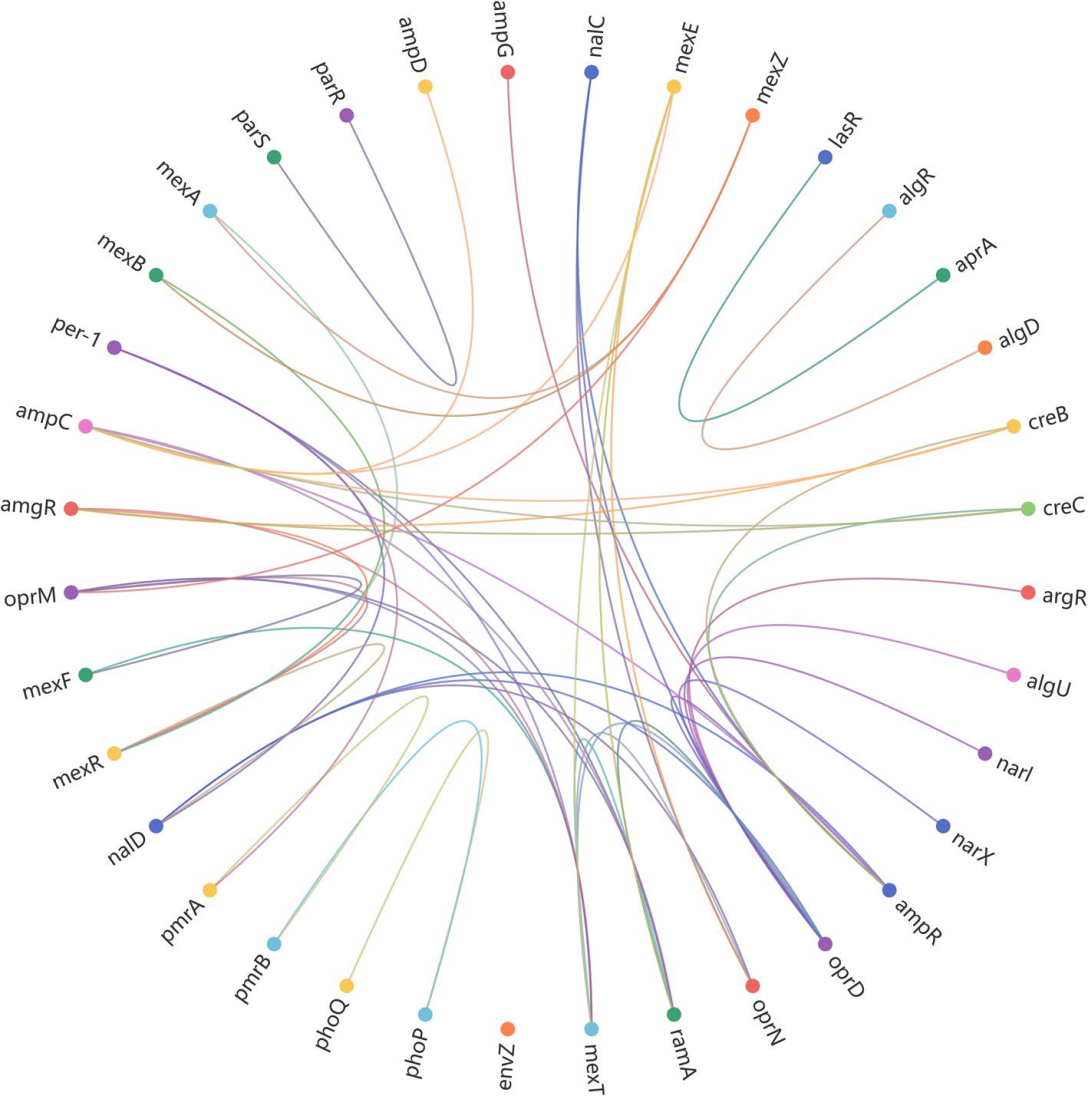
Potential targets in the regulation of drug resistance in PA

The analysis of outer membrane channel proteins, active efflux systems, genes encoding antibiotic-inactivating enzymes, and biofilm regulatory networks in PA identifies shared targets within the regulatory pathways associated with diverse mechanisms of drug resistance, as illustrated in Figs. 4 and 5. Key regulatory genes, including *mexT, ampR, argR, lasR, algT, creB, creC, nalD,* and *mexR*, function as pivotal nodes, orchestrating a wide array of downstream genes and metabolic pathways, which ultimately contribute to the development of drug resistance in PA.

## Discussion

We have summarized the genes and regulatory factors involved in four drug resistance pathways of PAand constructed a resistance network. The results indicate that the development of drug resistance in PA is the combined result of the regulation by multiple genes and pathways. Through the analysis of the resistance network, we found that the genes *mexT, ampR,* and *argR* exhibit regulatory control over multiple genes and resistance pathways, suggesting that they may serve as targets for the development of new antibiotics. We believe that by inhibiting the regulation of these targets, it may be possible to reverse the drug resistance of PA. MexT influences the expression of major multidrug resistance pump operon genes, including MexAB-OprM, MexCD-OprJ, MexEF-OprN, and MexXY, facilitating the expulsion of antibiotics from the cell and reducing intracellular antibiotic concentrations—a critical mechanism for acquiring multidrug resistance [104]. Beyond multidrug resistance pump regulation, MexT modulates over 100 genes associated with bacterial pathogenicity, adaptability, and survival [201]. For instance, MexT promotes the expression of the *oprD* gene, increasing the permeability of the PA outer membrane [202]. These intricate regulatory mechanisms confer bacteria with multiple resistances and sustained proliferation capabilities.

AmpR is a crucial regulatory element primarily responsible for controlling the activation of genes linked to β-lactam resistance [202]. It can detect and react to alterations in the bacterial cell wall, triggering a sequence of defensive responses. When the production of the cell wall is obstructed, AmpR becomes active, leading to the upregulation of genes like *ampC*, which enhances the cell's capacity to break down β-lactam medications and diminishes the drug's harmful effects on the cell [203]. Simultaneously, AmpR establishes a feedback mechanism with *mexR* to jointly oversee the expression of the MexAB-OprM pump [109]. Their collaborative efforts empower bacteria to develop a wider range of drug resistance. Apart from the aforementioned pivotal regulatory components, the transcription factor ArgR, associated with amino acid metabolism, also plays a crucial role in the development of resistance in PA. Detailed investigations have revealed that ArgR can directly influence the expression of the *oprD* gene, thereby impacting the cell's resilience to antibiotics [204]. Strains with a mutation deleting *argR* often exhibit heightened sensitivity to various inhibitors of amino acid synthesis.

Space limitations prevented us from covering all relevant secondary metabolites. Drug resistance trends in certain regions underrepresented due to the availability of data. Future research endeavors should be directed towards several pivotal objectives: conducting thorough investigations into the functions of drug-resistant genes utilizing state-of-the-art gene-editing technologies; employing systems biology methodologies to decipher the intricate network underpinning drug-resistance mechanisms; and pioneering the development of innovative antibiotics and alternative therapeutics to address the burgeoning crisis of antimicrobial resistance. Concurrently, reinforcing hospital infection control measures and enhancing public awareness on antibiotic resistance are pivotal in curbing the dissemination of drug-resistant strains [205]. Addressing the challenge of drug resistance in PA necessitates global collaboration and interdisciplinary efforts. Through the synergistic progress of basic research, clinical investigations, and public health policies, it is envisioned that this challenge can be more effectively tackled to safeguard humanity from the menace of drug-resistant strains [206].

## Conclusions and perspectives

Through analysis of the PA drug resistance regulatory network over the past 45 years, we have summarized that In PA, the *mexT* gene regulates both the cell membrane permeability and active efflux system resistance pathways, the *ampR* gene regulates both the cell membrane permeability and antibiotic inactivating enzyme resistance pathways, and the *argR* gene regulates both the cell membrane permeability and active efflux system resistance pathways. pivotal regulatory genes such as *mexT, ampR,* and *argR* are essential for the drug resistance mechanisms of PA and various physiological functions. These genes orchestrate the expression of multiple downstream effector genes, granting the bacteria diverse resistances, robust adaptability to the environment, and a competitive advantage for survival. Consequently, these central regulatory genes hold crucial promise and are anticipated to serve as promising targets for novel antibiotic development. Precision targeting of these regulatory elements to disrupt their interactions with downstream genes could potentially impede the emergence of bacterial resistance and pathogenicity.

The realization of targeted antibacterial strategies outlined above is a complex endeavor that necessitates comprehensive fundamental research on these regulatory genes and their molecular mechanisms.Regarding clinical interventions, prudent antibiotic usage, the innovation of new antimicrobial agents, and the application of gene-editing tools to directly target drug-resistant genes represent potential strategies to combat drug resistance. The CRISPR-Cas9 system has emerged as a potent toolkit for elucidating the roles of specific genes in conferring resistance [207]. Through the CRISPR-Cas9 system, we can design specific guide RNAs (gRNAs) that target genes associated with drug resistance inPA, such as efflux pump genes or beta-lactamase genes. By employing CRISPR-Cas9 to disrupt these genes, we are able to delineate their contributions to antibiotic resistance and investigate potential strategies for suppressing their expression. Furthermore, this system can be harnessed to generate precise PA mutant strains, facilitating the study of the emergence and dissemination of drug resistance. By comparing the susceptibility of wild-type and mutant strains to antibiotics, researchers can better understand how resistance emerges within bacterial populations. In addition to knockout experiments, CRISPR-Cas9 can also be used to activate or enhance the expression of specific genes to understand their roles in drug resistance [207]. This "gene activation" technique can help us identify genes that play a positive role in resistance, providing clues for the development of new therapeutic strategies. Furthermore, the application of systems biology approaches, such as constructing dynamic models of gene regulatory networks and metabolic pathways, offers new perspectives for understanding resistance mechanisms at a systems level and aids in the identification of new therapeutic targets [208].

In forthcoming research endeavors, we anticipate delving deeper into the regulatory mechanisms of antibiotic resistance genes inPA, employing interdisciplinary approaches to comprehensively understand the complexity of drug resistance. This encompasses the development of novel therapeutic strategies, such as specific inhibitors and gene-editing technologies, as well as investigating alternative therapies to conventional antibiotics, including antimicrobial peptides and immunomodulators. Concurrently, we are committed to translating these research findings into clinical practice, enhancing treatment outcomes through rapid diagnostic tools and personalized treatment plans. Moreover, strengthening hospital infection control and shaping public health policies will be focal points of our research, aiming to curtail the spread of drug-resistant strains and safeguard public health. Global collaboration and data sharing will be pivotal in addressing the issue of antimicrobial resistance, while raising awareness among healthcare professionals and the general public regarding the judicious use of antibiotics and the implications of resistance is equally crucial. Lastly, establishing long-term monitoring and evaluation mechanisms will ensure the continuous improvement and effectiveness of our control measures in confronting the challenges of antimicrobial resistance.

## Data Availability

Not applicable. All data was obtained from published studies and it is included in the manuscript.
